# Asian Variant Intravascular Lymphoma: A Look at the Laboratory Data of Lactase Dehydrogenase and Soluble Interleukin-2 Receptor

**DOI:** 10.7759/cureus.50243

**Published:** 2023-12-09

**Authors:** Eiichi Kakehi, Kazuhiko Kotani

**Affiliations:** 1 Department of General Medicine, Tottori Municipal Hospital, Tottori, JPN; 2 Division of Community and Family Medicine, Jichi Medical University, Shimotsuke, JPN

**Keywords:** soluble interleukin-2 receptor, lactase dehydrogenase, syndrome of inappropriate selection of antidiuretic hormone, hyponatremia, intravascular lymphoma

## Abstract

Asian variant intravascular lymphoma (AIVL) is a rare type of intravascular lymphoma that occurs in Asian populations. Syndrome of inappropriate antidiuretic hormone selection (SIADH) frequently occurs in patients with AIVL. Because it remains difficult to diagnose and has a poor prognosis, markers for early diagnosis are required. Although lactase dehydrogenase (LD) and soluble interleukin-2 receptor (sIL-2R) are diagnostic candidates, these markers do not appear to have been used often in prior studies. We present the case of an 87-year-old Japanese man with AIVL complicated by unexplained SIADH with a complaint of anorexia. Computer tomography showed splenomegaly but no lymphadenopathy. Elevated LD and sIL-2R were detected in the blood. The patient was diagnosed with AIVL through a random skin biopsy and was successfully treated with chemotherapy. When a patient presents with SIADH, we should actively look at or measure blood LD and sIL-2R for early diagnosis of ALVL. Further cases are warranted to determine these observations.

## Introduction

Intravascular large B-cell lymphoma is classified into three variants: the classical variant (with non-specific symptoms, unknown fever, and involvement of the central nervous system and skin); the cutaneous variant (with predominantly cutaneous findings); and the hemophagocytic-associated variants (with hemophagocytic syndrome, hepatosplenomegaly, and cytopenia) [1]. Hemophagocytic syndrome-associated intravascular lymphoma is found primarily in Asian populations and is also known as Asian variant intravascular lymphoma (AIVL) [1]. AIVL is a rare pathological condition and known to be difficult to diagnose because not only does it form extravascular tumor masses, but the diagnostic clues are apparently unestablished [1]. Because AIVL is more prevalently complicated by a syndrome of inappropriate antidiuretic hormone selection (SIADH) (66.7% of AIVL patients) than lymphoma (6.8%), it can have a late diagnosis, while the causes of SIADH are explored [2]. Although lactase dehydrogenase (LD) and soluble interleukin-2 receptor (sIL-2R) in the blood are generally thought to be useful markers for diagnosing lymphomas, these markers might be overlooked for AIVL; indeed, prior reports on AIVL complicated by SIADH have diagnosed AIVL post-mortem without the full utility of LD or sIL-2R [3-6]. Thus, it is necessary to emphasize cases that are diagnosed through the detection of LD and sIL-2R. Here, we present such a case of AIVL complicated by SIADH.

## Case presentation

An 87-year-old Japanese man was admitted to our hospital complaining of anorexia for the previous 3 weeks. He had a history of gastric cancer for which he had undergone surgical treatment at the age of 70 years and had not experienced recurrence since. He had not received any regular medication. The patient had no fever (temperature of 35.7°C), weight loss, or night sweats. His vital signs were normal. Physical examination revealed no specific findings, such as superficial lymphadenopathy or skin findings.

The results of the blood investigations on admission are presented in Table 1. Blood investigations revealed hyponatremia and elevated LD. Low blood osmolality, high urine osmolality, and high urinary sodium were detected with normal renal function. Endocrine examination showed normal adrenocorticotropic hormone (ACTH), cortisol, renin, aldosterone, thyroid-stimulating hormone, and thyroid hormone. Antidiuretic hormone (ADH) was elevated despite hyponatremia and low serum osmolality, indicating SIADH. Computer tomography showed splenomegaly (spleen index=508>480, 9.3 cm craniocaudal dimension × 9.1 cm width × 6.0 cm thickness) without solid tumors or other lymphadenopathies [7]. After admission, the patient’s condition deteriorated severely for one week. Because bicytopenia (hemoglobin, 7.0 g/dL; platelets, 4 x 10^4^/µL) was observed, along with decreased fibrinogen (101 mg/dL), increased fibrin degradation products (14.6 µg/mL), and a prolonged international normalized ratio of prothrombin time (1.40), the patient was diagnosed with disseminated intravascular coagulation (DIC) [8]. We suspected the patient had hemophagocytic syndrome. Subsequently, when measuring its relevant markers, we found a further increase in LD (480 U/L) and ferritin and sIL-2R levels. We considered some inflammatory and hematological disorders (i.e., lymphoma) to be SIADH.

**Table 1:** Blood and urinary investigations on admission WBC: White blood cell; RBC: Red blood cell; MCV: Mean corpuscular volume; TSH: Thyroid stimulation hormone; ACTH: Adrenocorticotropic hormone; ADH: Antidiuretic hormone; PT-INR: International normalized ratio of prothrombin time; APTT: Activated partial thromboplastin time; FDP: Fibrin degradation products; sIL-2R: Soluble interleukin-2 receptor; AST: Aspartate aminotransferase; ALT: Alanine aminotransferase; γGTP: γ-glutamyltransferase; LD: Lactase dehydrogenase; ALP: Alkaline phosphatase; BUN: Blood urea nitrogen

		Range			Range
Complete blood cell count			Tumor marker		
WBC, /µL	8,800	3,500–9,700	Ferritin, ng/mL	974	3.0–210
Neutrophil count, /µL	7,084	‐	sIL-2R, U/mL	7320	122–496
Basophile count, /µL	0.0	‐	Biochemical examination		
Eosinophile count, /µL	44	‐	Total protein, g/dL	6.0	6.5–8.2
Lymphocyte count, /µL	440	‐	Albumin, g/dL	3.6	3.7–5.5
Monocyte count, µL	1056	‐	AST, IU/L	35	10–40
Blast, %	0.0	‐	ALT, IU/L	20	5–45
RBC, /µL	438 x 10^4^	438–577	γGTP, IU/L	19	0–79
Hemoglobin, g/dL	13.4	13.6–18.3	LD, IU/L	324	124–222
MCV, fL	86	83–101	ALP, IU/L	113	38–113
Platelet, /µL	11 x 10^4^	14.0–37.9	Amylase, U/L	50	39–113
Immunoserological examination			Creatine kinase, IU/L	35	50–230
C-reactive protein, mg/dL	1.27	<0.30	BUN, mg/dL	18.4	8–20
Endocrinological examination			Creatinine, mg/dL	0.84	0.65–1.09
TSH, µIU/mL	0.70	0.5–5.0	Sodium, mEq/L	121	135–145
Free T_4_, ng/dL	0.82	0.9–1.7	Potassium, mEq/L	4.6	3.5–5.0
ACTH, pg/mL	16.9	7.2–63.3	Chlorine, mEq/L	87	98–108
Cortisol, µg/dL	11.96	6.24–18.00	Uric acid, mg/dL	4.0	0–7
Aldosterone, pg/mL	149	35.7–240	Glucose, mg/dL	98	70–109
Renin, pg/mL	7.1	2.5–21.4	Total cholesterol, mg/dL	109	150–219
ADH, pg/mL	75	<2.8	Triglyceride, mg/dL	129	50–149
Coagulation examination			Serum osmolarity, mOsm	250	286–292
PT-INR	1.40	0.90–1.13	Urinary examination		
APTT, second	45.9	26.0–38.0	Urinary osmolality, mOsm	666	‐
Fibrinogen, mg/dL	101	170–410	Urine sodium, mEq/L	102	‐
FDP, µg/mL	14.6	<5.0			

**Figure 1:**
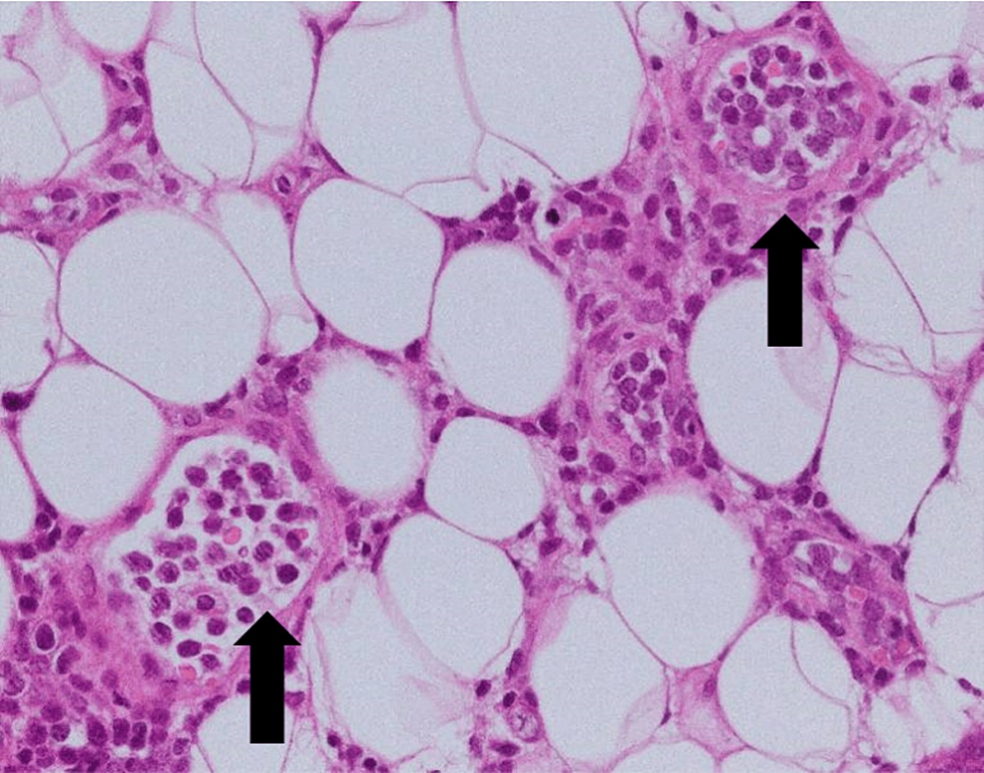
Pathological findings of random skin biopsies (hematoxylin and eosin staining) In the hematoxylin and eosin staining, the hyperplastic image of sporozoite-poor, medium to large, atypical, and similarly round cells was observed in small vessels of subcutaneous connective tissue (arrow).

**Figure 2:**
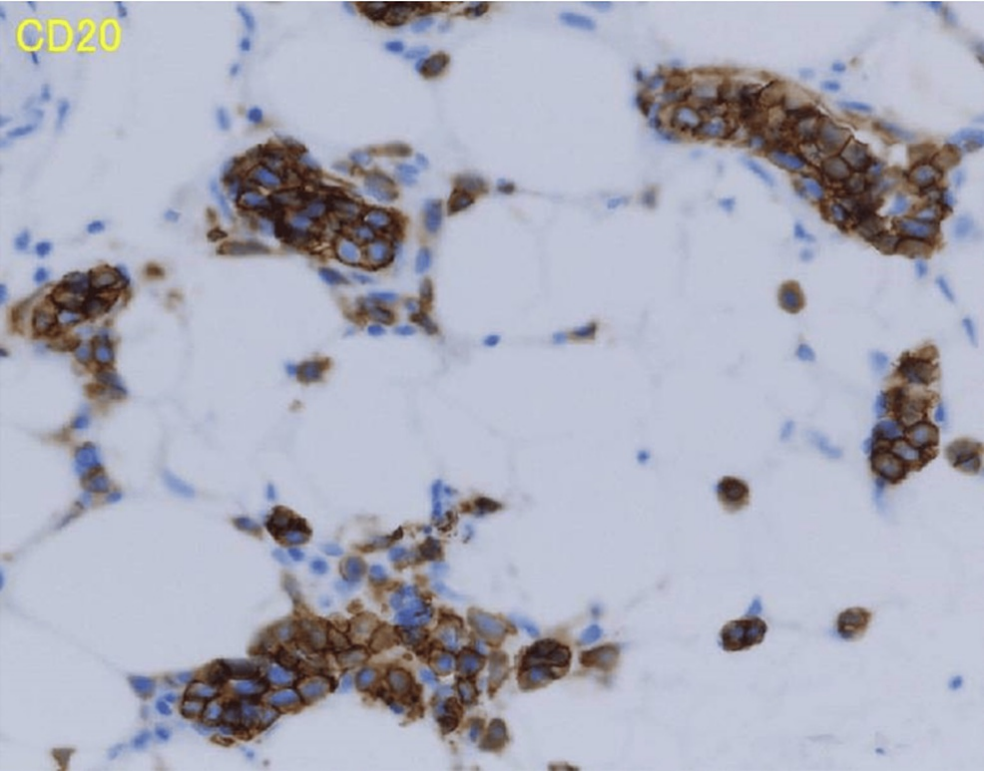
Pathological finding of random skin biopsy (immunohistochemical staining) Immunohistochemical staining showed CD20-positive cells and no CD3, CD5, or CD10 immune activity.

**Figure 3:**
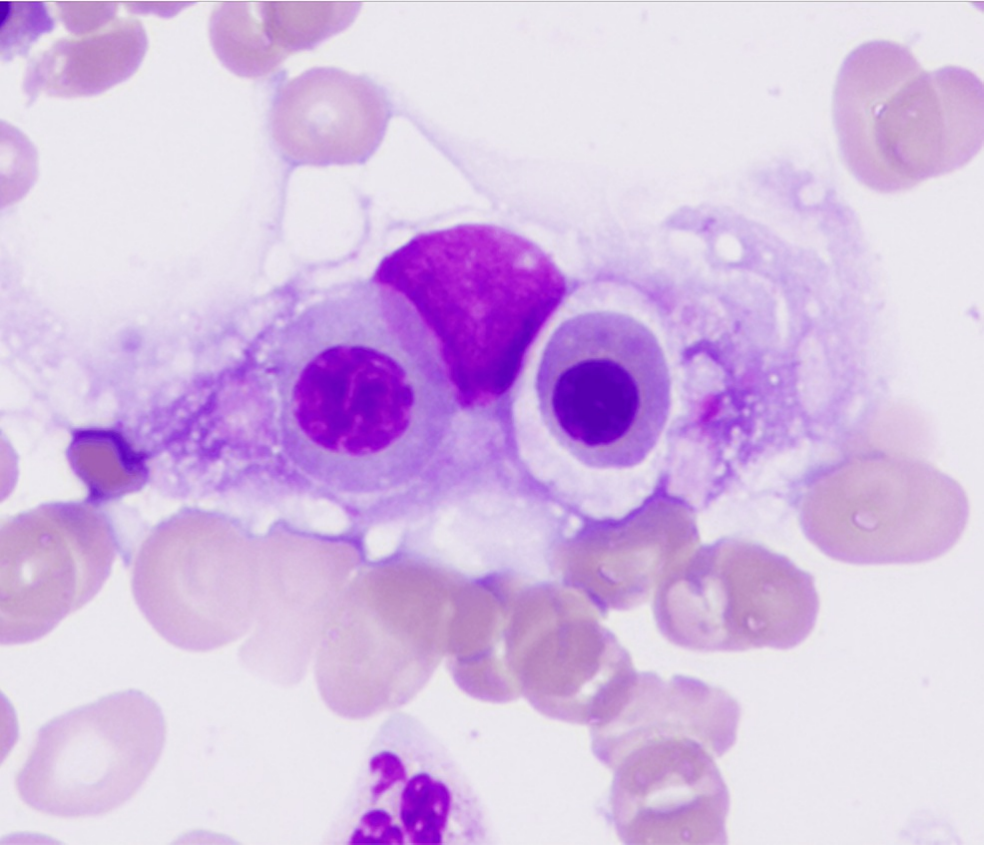
Pathological findings of bone marrow examination (hematoxylin and eosin staining) Hematoxylin and eosin staining showed hemophagocytosis by histiocytes.

Because the patient had no obvious skin findings, we performed random skin biopsies from the chest, abdomen, and right medial thigh. The histopathological findings revealed lymphoma cells with a large B-cell phenotype in blood vessels (Figure 1, 2). Bone marrow examination revealed hemophagocytosis by histiocytes (Figure 3). The patient was finally diagnosed with AIVL complicated by SAIDH. The patient received two courses of chemotherapy and was discharged after a two-month hospitalization. Both clinical symptoms and serum sodium levels improved owing to the treatment.

## Discussion

To improve the especially poor prognosis of AIVL, delayed early diagnosis must be avoided [4-6]. When searching the causes of SIADH, we first noted the changes in elevated LD, in addition to high sIL-2R levels. Following the notion that these markers could indicate a potential sign of intravascular lymphoma accompanied by SIADH, we were able to diagnose AIVL immediately. This seems to be an unusual case that was successfully treated over a short period. When we encounter an unexplained SIADH, it is necessary to look at or measure LD and sIL-2R for AIVL.

Patients with hemophagocytic syndrome often present a high blood LD and high sIL-2R level [9,10]. This syndrome can also be complicated by hyponatremia such as SIADH [11,12]. Because AIVL is described as a hemophagocytic syndrome-associated variant of intravascular lymphoma, it is reasonable that AIVL shows a similar clinical picture to hemophagocytic syndrome [1]. Thus, AIVL is a candidate pathology to be suspected when we encounter patients with hemophagocytic syndrome having elevated LD and sIL-2R or hyponatremia.

Blood LD levels are elevated in AIVL [3]. Tumor lysis is more likely to disintegrate when tumor cells are intravascular than when they are present as a mass, implying more elevated LD levels than general types of lymphomas [3]. Such patients can also have DIC, as in our case, resulting in elevated LD due to cell damage and necrosis [3]. Blood sIL-2R levels are elevated when matrix metalloproteinase-9 and tumor-associated macrophages induce the generation of sIL-2R and the cleavage of IL-2Rα-chains on the plasma membrane [13,14]. Furthermore, blood sIL-2R levels greater than 5,000 U/mL, as in our case, are implicated as being definitive of AIVL because sIL-2R elevation is greater in intravascular pathology, such as in DIC or phagocytic syndrome or a reaction associated with SIADH in AIVL rather than in general lymphomas [2,15]. Even though LD and sIL-2R are usually helpful markers for diagnosing lymphomas, they may be more strongly recognized for diagnosing AIVL.

## Conclusions

While AIVL shows a variety of clinical signs, especially hemophagocytic syndrome, this pathology can be complicated by SIADH. When a patient presents with unexplained SIADH, we should look at or measure blood LD and sIL-2R for the diagnosis of ALVL. Further studies in similar cases are warranted to determine this theory.
